# Mbt/PAK4 together with SRC modulates N-Cadherin adherens junctions in the developing *Drosophila* eye

**DOI:** 10.1242/bio.038406

**Published:** 2019-03-15

**Authors:** Stephanie M. Pütz

**Affiliations:** Institute of Medical Radiation and Cell Research, University of Würzburg, Biozentrum, Am Hubland, D-97074 Würzburg, Germany

**Keywords:** *Drosophila*, Eye development, p21-activated kinase Mbt/PAK4, SRC, N-Cadherin, Adherens junction

## Abstract

Tissue morphogenesis is accompanied by changes of adherens junctions (AJ). During *Drosophila* eye development, AJ reorganization includes the formation of isolated N-Cadherin AJ between photoreceptors R3/R4. Little is known about how these N-Cadherin AJ are established and maintained. This study focuses on the kinases Mbt/PAK4 and SRC, both known to alter E-Cadherin AJ across phyla. *Drosophila* p21-activated kinase Mbt and the non-receptor tyrosine kinases Src64 and Src42 regulate proper N-Cadherin AJ. N-Cadherin AJ elongation depends on SRC kinase activity. Cell culture experiments demonstrate binding of both *Drosophila* SRC isoforms to N-Cadherin and its subsequent tyrosine phosphorylation. In contrast, Mbt stabilizes but does not bind N-Cadherin *in vitro*. Mbt is required in R3/R4 for zipping the N-Cadherin AJ between these cells, independent of its kinase activity and Cdc42-binding. The *mbt* phenotype can be reverted by mutations in *Src64* and *Src42*. Because Mbt neither directly binds to SRC proteins nor has a reproducible influence on their kinase activity, the conclusion is that Mbt and SRC signaling converge on N-Cadherin. N-Cadherin AJ formation during eye development requires a proper balance between the promoting effects of Mbt and the inhibiting influences of SRC kinases.

## Abstract (precis)

**Summary:** N-Cadherin adherens junction formation in the *Drosophila* larval eye imaginal disc is controlled by the combined functions of the p21-activated kinase Mbt/PAK4 and the kinases Src64 and Src42*.*

## INTRODUCTION

Tissue morphogenesis in animals is accompanied by changes in composition of the zonula adherens (ZA) allowing dynamic intercellular interactions. In developmental processes, ZA becomes manifold reorganized e.g. during epithelial-to-mesenchymal transition (Lamouille et al., 2014; Schafer et al., 2014), or during *Drosophila* compound eye development (Tepass and Harris, 2007).

Adherens junctions (AJ) are a main adhesive complex of the ZA. The central molecules of AJ, the classical cadherins such as E-Cadherin (E-Cad) and N-Cadherin (N-Cad), establish intercellular homophilic interactions. The intracellular domain of Cadherin binds to Catenins, thereby linking the AJ to the actin cytoskeleton (Halbleib and Nelson, 2006; Harris, 2012). Cadherin levels at the cell membrane can be regulated by several distinct mechanisms, including gene expression, trafficking, protein turnover, post-translational modifications and protein degradation (Brown et al., 2006; Fujita et al., 2002; Loyer et al., 2015; Shindo et al., 2008). Besides regulation of cadherin levels, a switch between different cadherin subtypes can induce morphological changes (Halbleib and Nelson, 2006). How these different mechanisms result in coordinated reorganization of AJ during development is not completely understood.

**Fig. 1.**
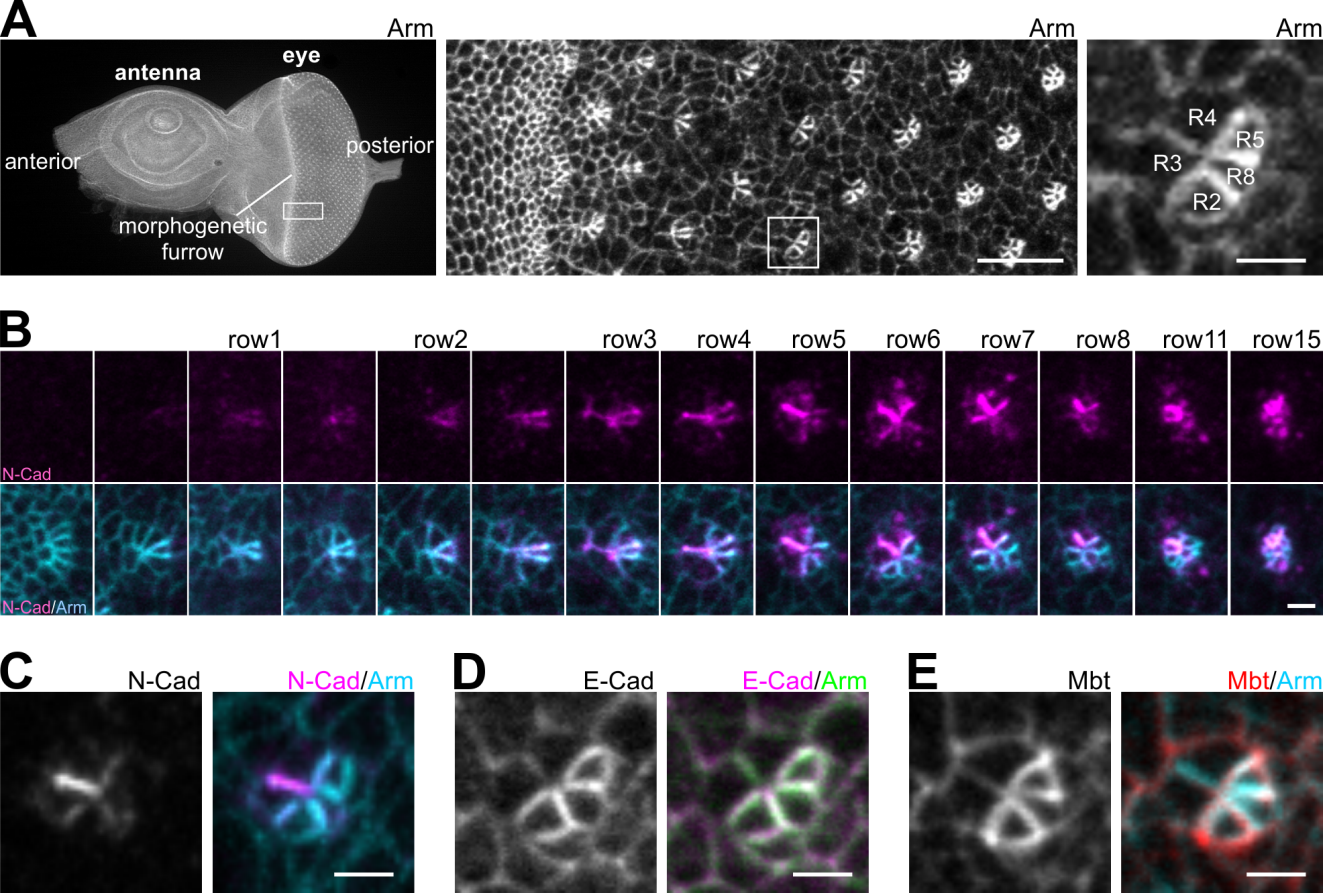
**Expression patterns of different AJ components in the larval eye disc.** (A) Overview of a complete wild-type eye disc, a detailed view of ommatidium formation posterior to the morphogenetic furrow (MF) and a row 5 ommatidium stained for Armadillo (Arm). (B) N-Cad (magenta) and Arm (cyan) staining of developing wild-type ommatidia ranging from the MF to row 15. N-Cad AJ zipping up the interface between R3 and R4 is observed between row 2 and 5. (C–E) Row 5 ommatidia stained for N-Cad (C), E-Cad (D) and Mbt (E) in combination with Arm. Scale bars: 10 µm and 2 µm for single ommatidia.

*Drosophila* eye imaginal discs are an easily accessible tissue to analyze AJ formation and reorganization necessary to finally establish the highly ordered arrangement of the 800 single eye units (ommatidia) in the adult compound eye. Eye discs are epithelia, from which all cell types of the eye are determined. Briefly, at the beginning of third larval stage, the morphogenetic furrow starts posterior and crosses the epithelium. Anterior to the morphogenetic furrow, cells proliferate and stick together by E-Cad AJ. Cells enter a G1 arrest and become determined posterior to the furrow in a stepwise fashion to build up ommatidia row by row (see also Fig. 1). First, photoreceptor cells R8 are determined, followed by R2/R5, R3/4, R1/6, R7 and non-neuronal cells (Baker, 2001; Kumar, 2012; Treisman, 2013). At the time point, when R8/R2/R5/R3/R4 are determined, E-Cad and N-Cad differentially accumulate at the interfaces of the photoreceptor cells (Tepass and Harris, 2007). E-Cad is enriched at the AJ of R2, R5 and R8. Although all photoreceptor cells express a minimum of N-Cad, only the AJ between R3 and R4 accumulate N-Cad (Mirkovic and Mlodzik, 2006). In this context, enrichment of E-Cad at the AJ, but not of N-Cad, depends on Rap1 signaling (Baril et al., 2014; O'Keefe et al., 2009). The mechanism of how N-Cad AJ between R3 and R4 are established and maintained is unknown.

Among the kinases involved in AJ dynamics is the non-receptor tyrosine kinase SRC (Lilien and Balsamo, 2005; Yeatman, 2004). SRC regulates E-Cad AJ stability through several different mechanisms. SRC activity has a bimodal effect on E-Cad AJ; low SRC activity levels are necessary for E-Cad cell adhesion and integrity of E-Cad cell–cell contacts, but high SRC activity disrupts E-Cad AJ (McLachlan et al., 2007; Shindo et al., 2008; Takahashi et al., 2005). In mammalian cells, SRC can induce ubiquitination of E-Cad and regulates E-Cad degradation (Fujita et al., 2002; Shen et al., 2008). In *Drosophila*, two SRC isoforms are encoded by two genes, *Src42* and *Src64*. Src42 localizes to E-Cad AJ and is found in a complex with E-Cad and Armadillo, the *Drosophila* homolog of β-Catenin (Shindo et al., 2008; Takahashi et al., 1996; Takahashi et al., 2005). E-Cad AJ stability and turnover during tracheal development is regulated by Src42 via a complex mechanism; Src42 activity on the one hand decreases E-Cad protein levels, but on the other hand increases E-Cad gene transcription (Shindo et al., 2008). Moreover, Src42 activity is elevated in cells undergoing morphogenesis (Shindo et al., 2008). Obviously the dual function of SRC and the fine tuning of its activity are important for tissue morphogenesis. During eye development, overexpression of Src42 or Src64 leads to E-Cad AJ defects and results in a rough-eye phenotype or complete eye absence (Langton et al., 2009; Pedraza et al., 2004).

The influence of SRC on N-Cad AJ has not been investigated in *Drosophila*. Overexpression of a mutated variant of Src42 had an influence on AJ between R3 and R4, but a potential impact on N-Cad was not analyzed (Takahashi et al., 1996). There are some hints from other organisms, e.g. in chicken lens epithelium, that inhibition or privation of active SRC from the N-Cad AJ is necessary to enable AJ maturation (Leonard et al., 2013). In human melanoma cells, phosphorylation of N-Cad by SRC is important for cell migration and affects N-Cad/β-Catenin complex stability (Qi et al., 2006).

A second kinase involved in AJ morphogenesis and dynamics is p21-activated kinase (PAK)4, termed Mushroom bodies tiny (Mbt) in *Drosophila* (Schneeberger and Raabe, 2003; Walther et al., 2016). PAKs are serine threonine kinases acting as downstream effectors of RhoGTPases. Depending on structural features and their mode of activation, they are subdivided into group I and group II PAKs (Jaffer and Chernoff, 2002; Kumar et al., 2017). Group I PAKs are activated by binding of their p21-binding domain (PBD) with a GTP-loaded RhoGTPase. PAK4/Mbt belongs to the group II PAKs and binding of GTP-loaded Cdc42 mainly serves as a localization determinant (Abo et al., 1998; Schneeberger and Raabe, 2003). Full stimulation of kinase activity apparently needs binding of further proteins. The SH3-domain of SRC has been discussed as such an activator (Ha et al., 2012; Ha et al., 2015; Kumar et al., 2017; Radu et al., 2014). Depending on cellular signaling, PAK4/Mbt can be found in the nucleus and cytoplasm, but primarily localizes at AJ (Jin et al., 2014; Li et al., 2012; Menzel et al., 2008; Schneeberger and Raabe, 2003). PAK4/Mbt presence in the cell is important for E-Cad AJ across phyla (Faure et al., 2005; Schneeberger and Raabe, 2003; Wallace et al., 2010; Walther et al., 2016) and Mbt function at the AJ is interlinked with Rap1-signaling and the apical determinant Par3/Bazooka (Walther et al., 2018). PAK4/Mbt kinase activity is required for formation and maturation of E-Cad AJ (Wallace et al., 2010; Walther et al., 2016). One mechanism of how PAK4/Mbt influences E-Cad AJ is phosphorylation of β-Catenin/Armadillo (Li et al., 2012; Menzel et al., 2008; Selamat et al., 2015; Walther et al., 2016). On one hand, Armadillo phosphorylation destabilizes the E-Cad/Armadillo complex in a cell culture model (Menzel et al., 2008). On the other hand, Armadillo phosphorylation by Mbt is important for Par3/Bazooka retention during ZA remodeling (Walther et al., 2016). Par3/Bazooka is necessary for membrane differentiation, but also for AJ maturation (Tepass, 2012). These opposing mechanisms enable E-Cad AJ remodeling and stability during eye development.

The expression of N-Cad during eye development raises the question of whether Mbt in addition to E-Cad also influences N-Cad AJ. In human glioma xenograft cells, strong overexpression of PAK4 increases N-Cad levels (Kesanakurti et al., 2017). Information about a direct link between Mbt and N-Cad AJ is missing. This paper addresses the question of whether the kinases Mbt/PAK4 and SRC influence the N-Cad AJ between photoreceptor R3 and R4.

## RESULTS AND DISCUSSION

### Differential localization of AJ components in the eye imaginal disc

Based on a previous report demonstrating the presence of N-Cad AJ at the cell boundary between photoreceptor R3 and R4 (Mirkovic and Mlodzik, 2006), we analyzed the expression and localization pattern of N-Cad during ommatidial assembly in late third-instar larvae. Using Armadillo (Arm)/β-Catenin as a general marker for AJ in the eye imaginal disc (Fig. 1A), expression of N-Cad is initially observed in photoreceptor R8 (row1 posterior to the morphogenetic furrow, Fig. 1B). In row 3, N-Cad becomes visible as a point like structure in the center of the ommatidial pre-cluster. With determination of R3/R4, N-Cad AJ elongate between these photoreceptor cells and N-Cad strongly accumulates particularly at this cell interface (row 5), which corresponds to earlier observations (Mirkovic and Mlodzik, 2006). In addition, weaker N-Cad signals can be detected between R2/R3 and R4/R5.

For comparative analysis of the AJ components E-Cad and Mbt, row 5 ommatidial pre-clusters were chosen because N-Cad AJ are fully elongated and prominently visible (Fig. 1C). E-Cad is seen at all AJ but accumulates at cell boundaries of R2 and R5 (Fig. 1D; Fig. S1A). Mbt localization pattern is similar to E-Cad (Fig. 1D,E), corresponding to the observation that Mbt is a main component of E-Cad AJ (Walther et al., 2016). Mbt staining at the R3/R4 interface is weaker and in several ommatidia nearly absent (Fig. 1E and data not shown). A second difference to E-Cad is a slightly more intense Mbt staining at cell–cell contacts between the ommatidial pre-cluster and surrounding non-differentiated cells, forming a ring around each cluster (Fig. 1E).

### Mbt is required for N-Cad AJ elongation

**Fig. 2.**
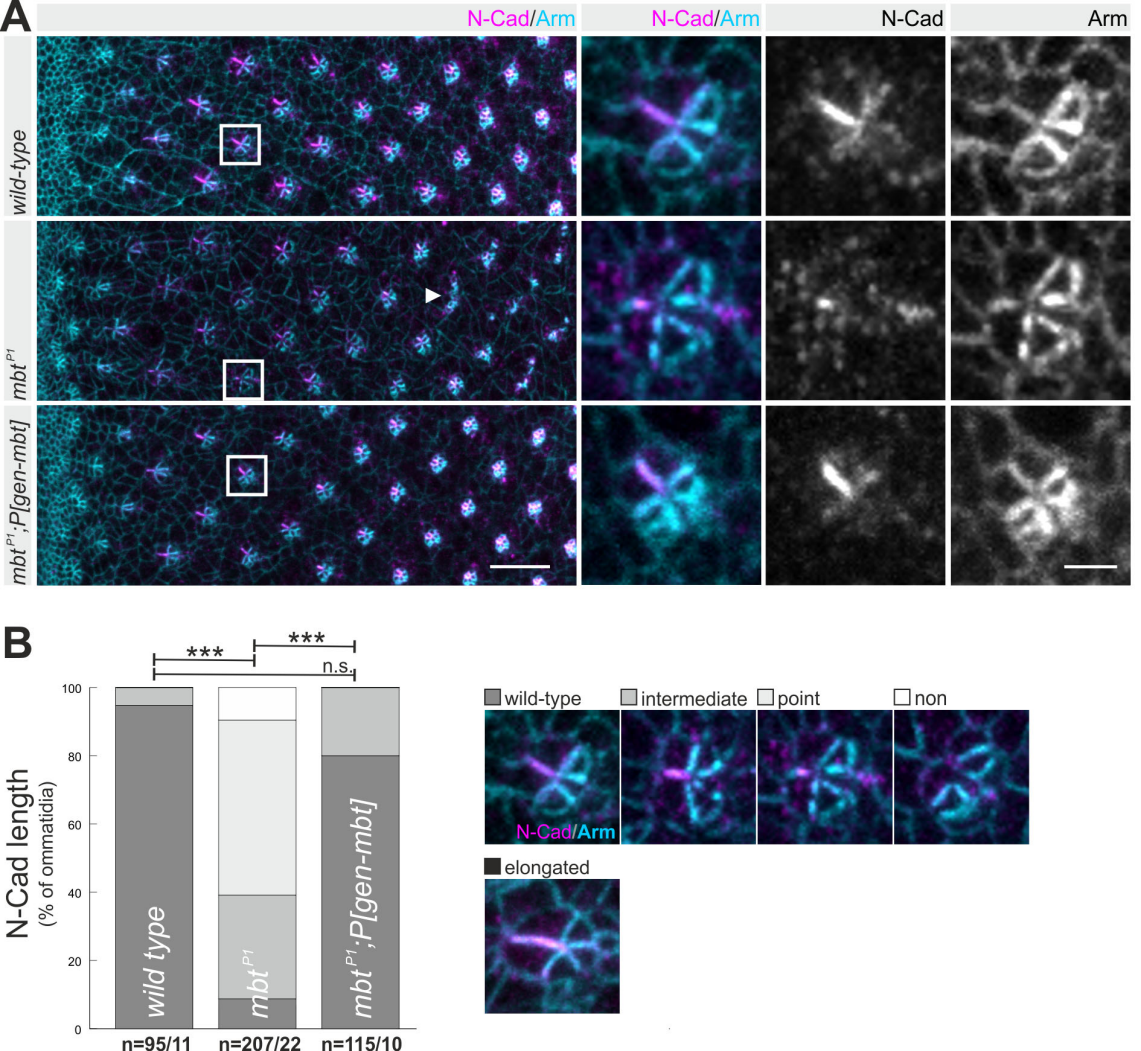
**Mbt is required for elongated N-Cad AJ between photoreceptor R3 and R4.** (A) Views from eye discs posterior to MF and representative row 5 ommatidia of the genotypes wild type*, mbt^P1^*, and *mbt^P1^;P[gen-mbt]* stained for N-Cad (magenta) and Arm (cyan). Squares indicate selected row 5 ommatidia and the arrowhead a non-cohesive row 11 *mbt^P1^* ommatidium. Scale bars: 10 µm and 2 µm, respectively. (B) Quantitative analysis of N-Cad AJ length. Displayed are the percentages of row 5 ommatidia showing a wild-type N-Cad AJ, a shortened N-Cad AJ (intermediate, point or non) or an elongated N-Cad AJ, classified according to the pictures on the right site. Asterisks indicate significance. *n*= number of ommatidia/number of eye discs analyzed, in this and all following graphs.

**Fig. 3.**
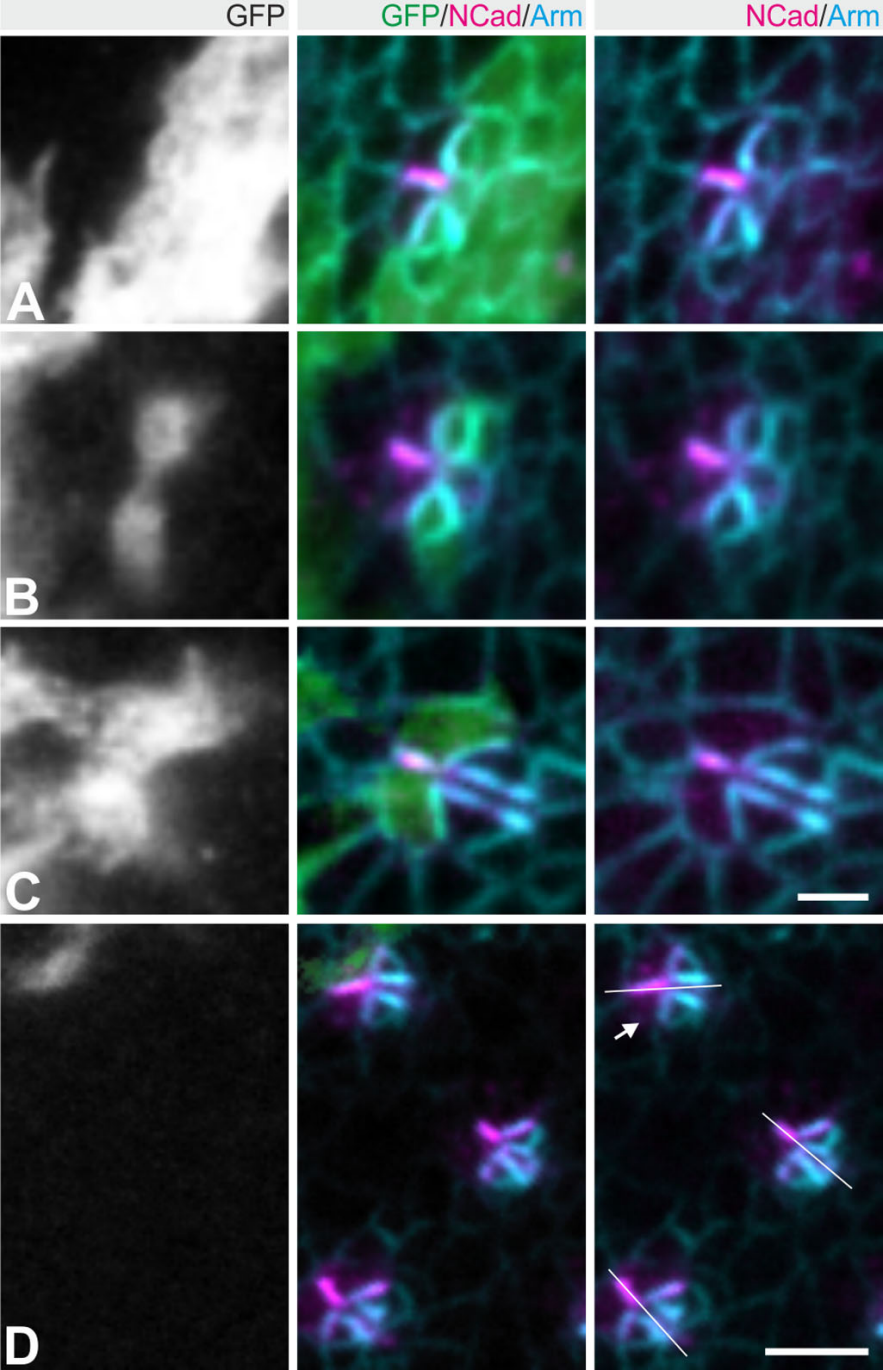
**Mbt mosaic analysis.** Representative row 5 ommatidia with loss of Mbt in R2/R5/R8 (A), R2/R5 (B) or R3/R4 (C) as indicated by expression of GFP (green). Eye discs were co-stained for N-Cad (magenta) and Arm (cyan). (D) Rotation defect in an ommatidium with a *mbt^P1^* R4 cell (arrow). The morphogenetic furrow is to the left and the equator to the bottom. White lines indicate the degree of rotation. Scale bars: 4 µm and 2 µm for single ommatidia.

The weak Mbt staining at the R3/R4 interface raises the question whether Mbt affects only E-Cad or also N-Cad AJ. Therefore, N-Cad staining was analyzed in *mbt^P1^* (null allele for *mbt*) larvae and compared to wild type and animals expressing a genomic rescue construct in the *mbt^P1^* background (*mbt^P1^;P[gen-mbt]*) (Fig. 2A). Posterior row 10, *mbt^P1^* ommatidia lose their cohesion (Schneeberger and Raabe, 2003) and N-Cad is often detected at the outer edges (arrowhead in Fig. 2A). More anterior in row 5, the phenotype seen in *mbt^P1^* is variable, ranging from complete absence of N-Cad staining to an apparently wild-type appearance. Most frequently, N-Cad is only seen as a point-like structure (Fig. 2A). Phenotypes were classified according to N-Cad length and displayed as percentage of all analyzed ommatidia (Fig. 2B). Quantification and statistical analysis of the respective phenotypic classes verified significant differences between wild type and *mbt^P1^*. The specific effect of Mbt on N-Cad was supported by analysis of *mbt^P1^;P[gen-mbt]* animals*.* The genomic construct completely reverted the N-Cad phenotype (Fig. 2A,B). This provided evidence that Mbt is required for assembly of elongated N-Cad AJ between photoreceptors R3 and R4. The specific requirement of Mbt in R3 and R4 for N-Cad AJ formation was analyzed by clonal analysis using the MARCM-technique (Lee and Luo, 1999). Homozygous *mbt^P1^* cells were detected by co-expression of the mCD8::GFP reporter. The majority (>87%, *n*=16) of ommatidia with a loss of Mbt only in R2, R5 and/or R8 showed wild-type-like N-Cad AJ (Fig. 3A,B). In contrast 90% of mosaic ommatidia with a short N-Cad AJ lack Mbt in R3 and/or R4 (*n*=29). Importantly, five out of six *mbt^P1^* clones only affecting R3/R4 but not R2/R5/R8 had a short N-Cad AJ (Fig. 3C). Interestingly, we also noticed differential effects in clones either effecting R3 or R4. Whereas short N-Cad AJ are more often observed when R3 lacks Mbt function, clones affecting R4 frequently showed rotation defects (Fig. 3D). As described earlier, N-Cad is important for proper ommatidial rotation (Mirkovic and Mlodzik, 2006). Whether Mbt influences the ommatidial rotation via N-Cad or another mechanism (e.g. Bazooka, see also below) remains an open question. In conclusion, the mosaic analysis revealed a requirement of Mbt in R3 and R4 for elongated and functional N-Cad AJ. This is the first description of a group II PAK relevant for N-Cad AJ formation. In contrast, mammalian group I PAKs are activated downstream of N-Cad AJ during myoblast differentiation (Joseph et al., 2017) and synapse remodeling (Xie et al., 2008).

Our findings also raised the question of whether Mbt influences differential expression of N-Cad and E-Cad. In pupal eye discs, the importance of Mbt for E-Cad AJ stability has been shown (Walther et al., 2016). In larval *mbt^P1^* eye discs, accumulation of E-Cad at the R2 and R5 AJ was impaired (compare Fig. S1A,B), a phenotype that could be reverted by expression of *P[gen-mbt]* (Fig. S1C). Impaired E-Cad accumulation in *mbt^P1^* might explain the loss of ommatidial integrity posterior row 10 (arrowhead in Fig. 2A). No upregulation of E-Cad was seen at the R3/R4 AJ in *mbt^P1^* (compare Fig. S1D,E,F), which is in line with the observation that E-Cad is not upregulated when N-Cad is absent (Mirkovic and Mlodzik, 2006).

Another important AJ modulator is Bazooka (Baz), which was shown in pupal eye discs to link Mbt to E-Cad AJ stability (Walther et al., 2016). In larval eye discs, Baz is specifically enriched in R3/R4 and later in R4 (Djiane et al., 2005; Fig. S2A). Following row 5 ommatidia from apical to more basal sections, Baz staining is first visible mainly in R4, followed by Baz localization at all photoreceptor boundaries and elevated Baz signal at the R3/R4 interface. Baz staining overlaps with N-Cad signals only in more basal sections (Fig. S2B), showing apical to basal polarity of apical determinants and ZA material. In *mbt^P1^* ommatidia, Baz showed weak, more diffuse, and uneven membrane localization (Fig. S2A). Comparable results were found in *mbt^P1^* pupal eye discs (Walther et al., 2016). Despite the general reduction in Baz staining intensity, enrichment of Baz specifically in R4 was visible at least to some degree (Fig. S2A, arrowhead). Since the loss of Mbt, specifically in R4, leads to ommatidial rotation defects (Fig. 3D) and apical determinants such as Baz regulate planar cell polarity (Djiane et al., 2005), it remains an open question whether Mbt influences ommatidial rotation via Baz.

### N-Cad AJ formation is independent of Mbt kinase activity

**Fig. 4.**
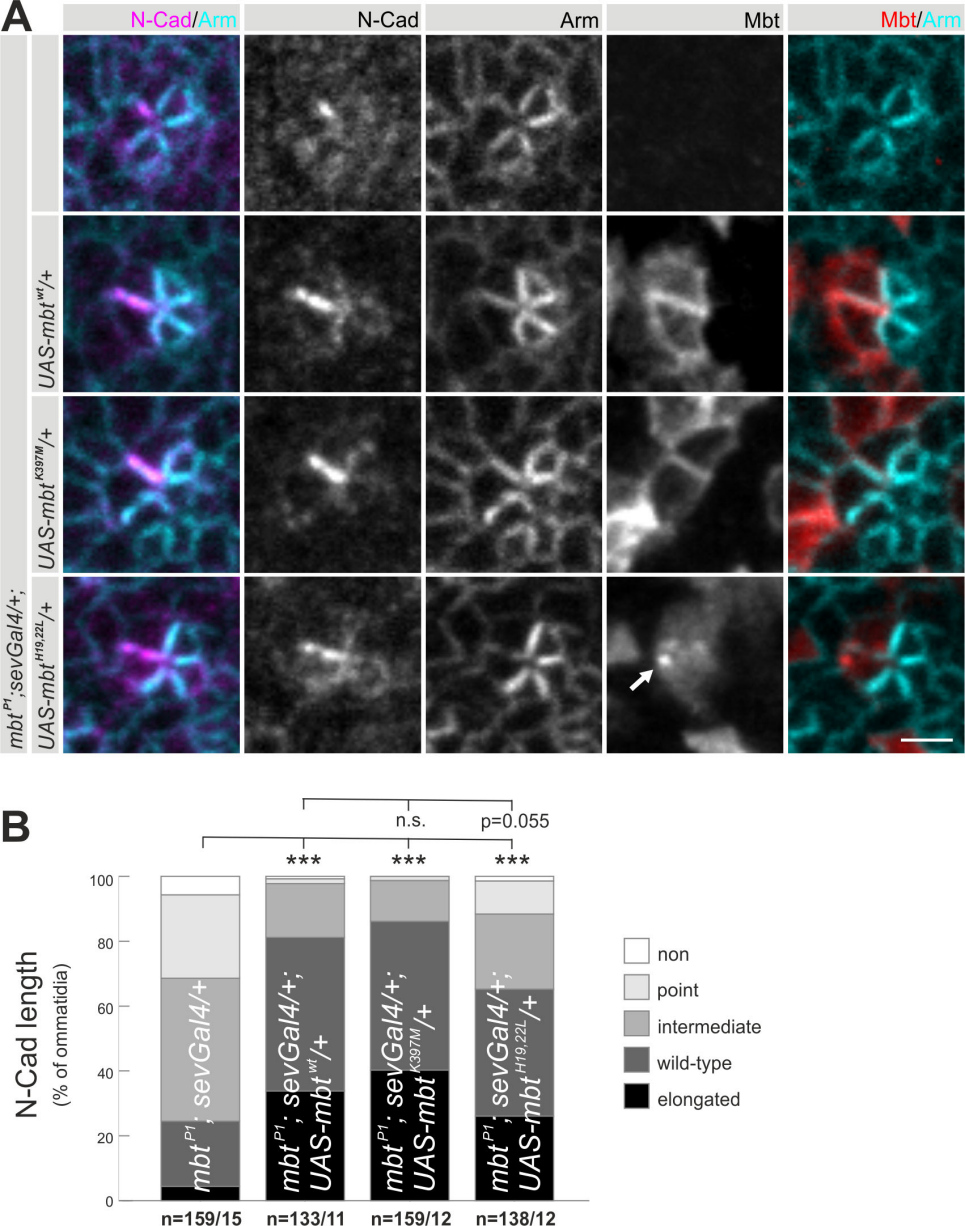
**N-Cad AJ elongation is independent of Mbt kinase activity.** (A) Representative row 5 ommatidia expressing Mbt^wt^, Mbt^K397M^ or Mbt^H19,22L^ under *sev-Gal4* control in an *mbt^P1^* background, stained for N-Cad (magenta), Arm (cyan) and Mbt (red). *mbt^P1^; sev-Gal4/+* ommatidia were used as control. The arrow indicates accumulation of Mbt^H19,22L^. Scale bar: 2 µm. (B) Quantitative analysis of N-Cad AJ length. Displayed are the percentages of ommatidia with N-Cad AJ lengths classified according to Fig. 2B. Asterisks indicate significance.

Baz together with Arm mechanistically links Mbt kinase activity to E-Cad AJ stability (Schneeberger and Raabe, 2003; Walther et al., 2016). Besides the C-terminal kinase domain, Mbt has a second well characterized structural domain: the N-terminal located binding site (PBD) for the RhoGTPase Cdc42, which is required for Mbt membrane localization as well for E-Cad AJ remodeling and stability (Melzer et al., 2013; Schneeberger and Raabe, 2003; Walther et al., 2016). This raised the question about the functional role of both domains with respect to N-Cad AJ. To address this point, *UAS-mbt* transgenes encoding a PBD-defective (Mbt^H19,22L^), a kinase-deficient (Mbt^K397M^) or a wild-type (Mbt^wt^) variant were expressed in the *mbt^P1^* background in photoreceptor R3 and R4 using *sev-Gal4*. Animals of the genotype *mbt^P1^;sev-Gal4/+* served as a control. N-Cad phenotypes were analyzed in row 5 ommatidia and quantified. Transgenic expressed Mbt^wt^ localizes to the cytosol and cell boundaries of the cells (Fig. 4A). As seen for the genomic *mbt* rescue construct (Fig. 2), expression of *UAS-mbt^wt^* reverted the N-Cad AJ to a wild-type appearance (Fig. 4A,B). Although *sev-Gal4* expression is not restricted to R3/R4 (Ray and Lakhotia, 2015), it again indicated that Mbt is required in R3 and R4 to build up N-Cad AJ between these cells. The kinase deficient Mbt^K397M^ predominantly localizes at the membrane. Interestingly, Mbt^K397M^ rescued the R3/R4 N-Cad AJ phenotype of *mbt^P1^* to a similar degree as the non-mutated transgene (Fig. 4A,B). Thus, kinase activity of Mbt is not necessary to establish and maintain N-Cad AJ. This is in contrast to E-Cad AJ, where Mbt kinase activity is needed for Armadillo phosphorylation, followed by Bazooka retention and finally E-Cad AJ stability and morphogenesis (Walther et al., 2016). For N-Cad AJ formation such a mechanism is unlikely, since the N-Cad AJ is independent of Mbt kinase activity, thus Armadillo phosphorylation by Mbt can't be the initial step. It remains an open question as to whether Armadillo phosphorylation by other kinases plays a role during N-Cad AJ formation. In previous studies, quantitative and qualitative differences for Armadillo binding to E-Cad and N-Cad were described. N-Cad is less efficient than E-Cad at sequestering Armadillo (Loureiro and Peifer, 1998; Schafer et al., 2014). This is also reflected by differential Armadillo staining during ommatidial assembly, showing weak signal at N-Cad AJ and high signal at E-Cad AJ (Fig. 1A,C,D).

To assess whether membrane localization of Mbt is needed for normal N-Cad AJ, the Cdc42-binding-deficient Mbt^H19,22L^ variant was used (Schneeberger and Raabe, 2003). Mbt^H19,22L^ staining was found in the cytosol, reflecting the relevance of RhoGTPase binding for Mbt membrane localization (Fig. 4A). Besides this, Mbt^H19,22L^ accumulated in point-shaped structures (arrow in Fig. 4A). Surprisingly, the *mbt^P1^* N-Cad AJ phenotype was significantly reverted by Mbt^H19,22L^, but rescue was not complete (Fig. 4A,B). Compared to the wild-type transgene, rescue of the phenotype with *mbt^H19,22L^* transgene shows a tendency to be different (Fig. 4B, *P*-value near the threshold of 0.05). To verify this result a second independent *UAS-mbt^H19,22L^* fly line was tested, showing again a N-Cad phenotype between *mbt^P1^* and wild type (data not shown).

The observation that Mbt staining is minimal at the R3/R4 cell–cell boundary (Fig. 1E), but nevertheless is required in a kinase-independent and presumably membrane localization independent manner for N-Cad AJ formation and elongation, raises the question of the functional relevant domains of Mbt mediating this effect and at which subcellular site Mbt is required. This parallels findings from other group II PAKs. For example, human PAK4 binds to and thereby protects RhoU from ubiquitination in a kinase activity and Cdc42 independent manner (Dart et al., 2015).

### Elevated SRC levels lead to truncated N-Cad AJ

A second kinase relevant for the R3/R4 AJ is the tyrosine kinase SRC (Takahashi et al., 1996). Both *Drosophila* SRC isoforms (Src42 and Src64) are expressed in larval eye discs (Kussick et al., 1993; Takahashi et al., 1996). Studies of other organisms indicate that active SRC destabilizes N-Cad AJ (Leonard et al., 2013) and induces β-Catenin phosphorylation at Y654, which is associated with N-Cad degradation (Zhou et al., 2011). Y654 and the adjacent sequence are conserved in Armadillo (Y667). Moreover, Qi and co-workers identified human N-Cad as a direct phosphorylation substrate of SRC and described Y860 as the major site relevant for β-Catenin dissociation from the N-Cad/β-Catenin complex (Qi et al., 2006). The comparison of N-Cad intracellular sequences from different organisms reveals conservation of Y860 across phyla (Fig. S3).

**Fig. 5.**
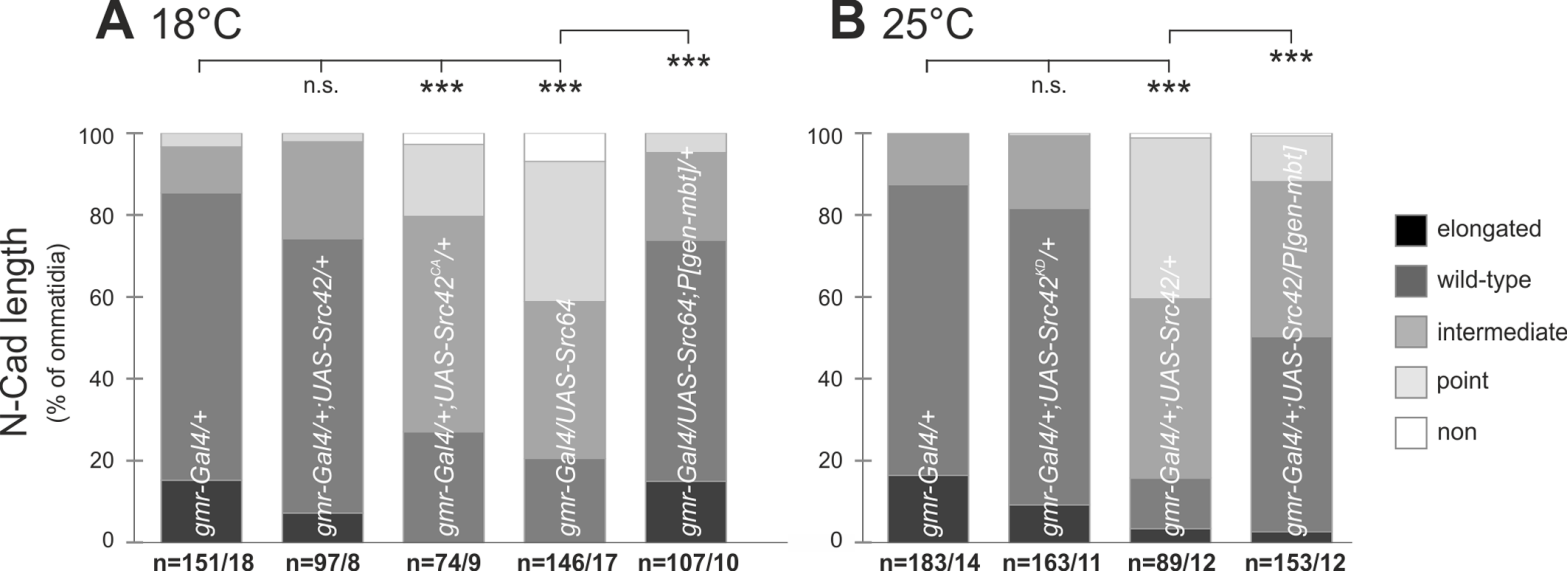
**Elevated expression of SRC isoforms shorten the N-Cad AJ between photoreceptor R3 and R4.** Quantitative analysis of N-Cad AJ length between R3 and R4. Displayed are the percentages of ommatidia with N-Cad AJ lengths classified according to Fig. 2B. Animals expressing *UAS-Src64*, *UAS-Src42*, *UAS-Src42^KD^* or *UAS-Src42^CA^* under *gmr-Gal4* control grown at 18°C (A) or 25°C (B), respectively. To test for genetic interaction, *UAS-Src64* and *UAS-Src42^CA^* were also analyzed in combination with *P[gen-mbt]*. *Gmr-Gal4/+* were used as control. For representative images, see Fig. S5. Asterisks indicate significance.

During chicken lens morphogenesis, SRC inactivation is necessary for maturation of N-Cad junctions, which are zipping up the cell–cell interface (Leonard et al., 2013). This zipper mode also reflects the observation at the *Drosophila* R3/R4 cell boundary (Fig. 1B). To investigate whether elevated, constitutively-active or kinase-deficient SRC disrupts the N-Cad AJ between R3 and R4, UAS-transgenic lines for Src42, constitutively active Src42^CA^, kinase deficient Src42^KD^ and Src64 were used. No crawling L3 larvae emerged upon expression of *UAS-Src42^CA^* and *UAS-Src64* with the *sev-Gal4* driver. Therefore, the driver-line was replaced by *gmr-Gal4* as in previous studies (Pedraza et al., 2004). Since over-expression of Src64 as well as Src42^CA^ at 25°C led to massive defects during eye development (Pedraza et al., 2004) (Fig. S4), larvae were grown at 18°C to reduce expression levels. Also at 18°C, expression of Src42^CA^ or Src64 interferes with eye development. However, the majority of row 5 ommatidia were intact but showed significantly truncated N-Cad AJ (Fig. 5A; Fig. S5A). Src42 did not exhibit such a strong phenotype at 18°C (Fig. 5A; Fig. S5A). Over-expression of Src42 at 25°C resulted in a fewer number of intact ommatidia and again in significantly truncated N-Cad AJ between R3 and R4 (Fig. 5B; Fig. S5B). In contrast, expression of Src42^KD^ did not alter the N-Cad AJ (Fig. 5B; Fig. S5B), demonstrating the requirement of a functional kinase domain to induce this phenotype. To sum up, SRC proteins impede N-Cad AJ maturation during *Drosophila* eye development. The mechanism by which Src42 affects N-Cad AJ is most likely kinase dependent, because Src42^CA^ had a much stronger influence compared to Src42, whereas Src42^KD^ had no effect. This is consistent with known SRC functions towards N-Cad in other organisms (Leonard et al., 2013; Qi et al., 2006).

### Src64 and Src42 genetically interact with Mbt to modulate N-Cad AJ

**Fig. 6.**
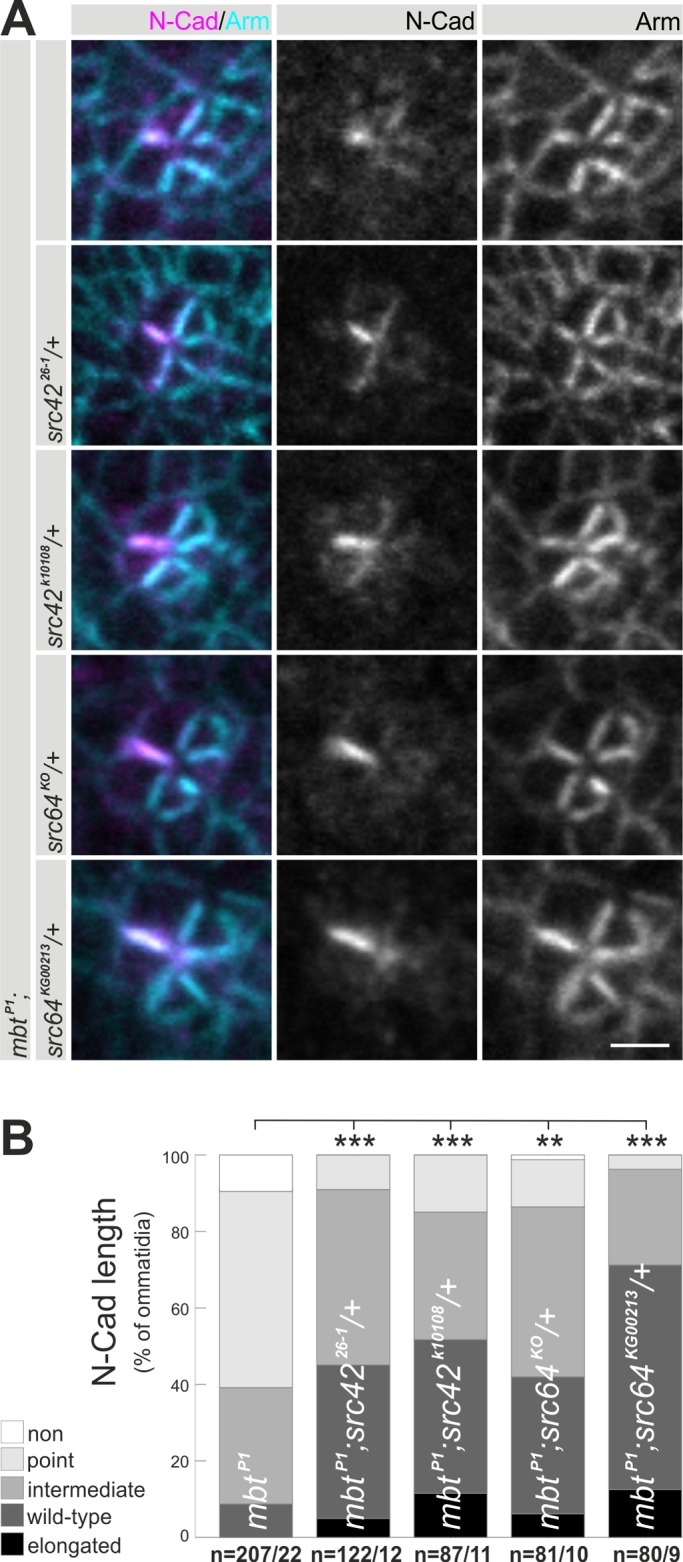
**Genetic interaction between *mbt* and *Src*.** (A) Representative single ommatidia from *mbt^P1^* males carrying in addition different heterozygous S*rc42* or *Src64* mutations stained for N-Cad (magenta) and Arm (cyan). Scale bar: 2 µm. (B) Quantitative and statistical analysis of the N-Cad AJ length confirmed significant suppression of the *mbt^P1^* N-Cad AJ phenotype by *Src* mutations Displayed are the percentages of ommatidia with N-Cad AJ lengths classified according to Fig. 2B. Asterisks indicate significance.

Both loss of Mbt and elevated SRC levels lead to truncated N-Cad AJ. This observation raises the question as to whether PAK4/Mbt and SRC act in concert or independently. To investigate a possible genetic interaction, N-Cad AJ in animals heterozygous for *Src64* or *Src42* mutations in an *mbt^P1^* background were examined. In all tested allelic combinations, the *mbt^P1^* N-Cad AJ phenotype was reverted back to a more wild-type appearance (Fig. 6A). Rescue of N-Cad AJ length was not complete, but significantly different to *mbt^P1^* (Fig. 6B). As a control, analysis of animals heterozygous for the different *Src* mutations in an otherwise wild-type background showed no influence on N-Cad AJ compared to wild type (Fig. S6). This finding argues against a function of SRC proteins upstream of Mbt in this cellular context. To substantiate the genetic interaction between SRC and Mbt, the reverse experiment was performed. Src42 or Src64 were overexpressed with *gmr-Gal4* in combination with the *P[gen-mbt]* transgene. Increased levels of Mbt significantly reverted the N-Cad phenotype caused by Src42 or Src64 overexpression to a more wild-type appearance (Fig. 5A,B; Fig. S5A,B). The genetic data suggest a function of *Drosophila* SRC either downstream of Mbt in the same pathway or in a parallel pathway that converges with Mbt signaling on N-Cad.

### Differences in the PAK4/Mbt-SRC signaling axis between *Drosophila* and other organisms

Following the hypothesis that both kinases act in the same pathway, Mbt should somehow act on SRC function. So far it is not clear whether Mbt/PAK4 and SRC undergo a direct protein–protein binding. However, evidence for bi-directional PAK4-SRC signaling comes from studies in vertebrates (Ha et al., 2012; Zanivan et al., 2013). Specifically, the isolated SH3 domain of human SRC activated human PAK4 (Ha et al., 2012). It was assumed that binding of the SRC SH3 domain to a RPKP-motive in PAK4 prevents intramolecular binding between the pseudosubstrate region and the kinase domain thereby resulting in loss of PAK4 auto-inhibition (Ha et al., 2015; Radu et al., 2014). In Mbt, the RPKP-motive is changed to RPLP (Fig. S7A), raising the question of whether Mbt is able to bind Src64 and Src42. To test this, Myc-tagged Mbt and His-tagged full length Src64 or Src42 were expressed in *Drosophila* S2 cells and co-immunoprecipitations were performed. No interaction was detected (data not shown). However, binding could be very transient or dependent on unknown signaling events influencing localization or accessibility of interaction domains. It remains to be verified whether PAK4/Mbt binds to full length SRC in any organism.

A second hint for direct PAK4-SRC signaling comes from an integrated analysis of phosphoproteomic data and prediction of kinase activities in a mouse skin carcinogenesis model (Zanivan et al., 2013). This study linked elevated PAK4 activity in malignant skin tumors with phosphorylation of serine 17 in mouse SRC, which together with phosphorylation of serine 12 by protein kinase C delta activated SRC (Gould and Hunter, 1988; Zanivan et al., 2013). This goes along with the observation in another study, where knockdown of PAK4 resulted in reduced SRC activity (Siu et al., 2010). Since kinase activity of Mbt is not required for N-Cad AJ elongation between R3/R4, phosphorylation is probably not the mechanistic link between Mbt and SRC regarding N-Cad AJ. This conclusion is supported by sequence comparison of mouse, human and *Drosophila* SRC proteins: the N-terminal SRC sequences containing S17 and S12 are highly conserved in vertebrates but absent in *Drosophila* Src64 and Src42 (Fig. S7B). To sum up, the relevant protein sequences with respect to the PAK4-SRC signaling in vertebrates are different or absent in *Drosophila*. Nevertheless, the genetic interaction between *mbt* and *Src64* or *Src42* under loss of function and overexpression conditions raises the question of whether Mbt regulates SRC activity. To examine this possibility, SRC activity in lysates from S2 cells was monitored by western blot with a phospho-Y416-Src antibody under normal and Mbt overexpression conditions. No reproducible influence was observed, neither on Src64 nor Src42 (*n*≥4, data not shown). To sum up, a direct molecular link between Mbt and SRC in *Drosophila* is unlikely.

### Mbt stabilizes N-Cad intracellular domain in cell culture experiments

**Fig. 7.**
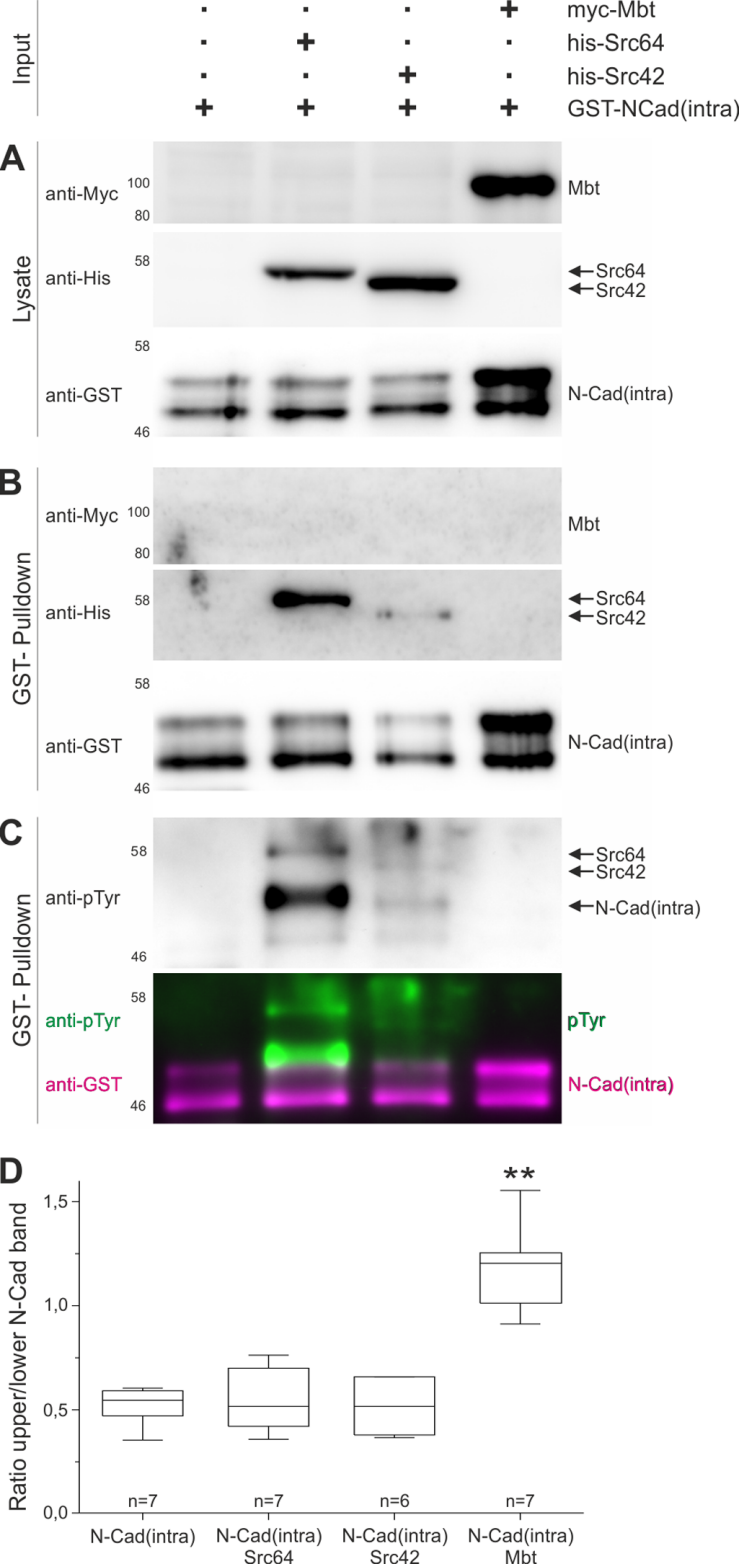
**Mbt and SRC signaling converge on N-Cad.** (A) Western blot analysis of lysates from stable S2 cell lines expressing GST-N-Cad(intra) alone or in combination with His-tagged Src64, Src42 or Myc-tagged Mbt. Blots were probed with anti-GST, anti-His or anti-Myc antibodies. (B) GST-pulldowns from the same original samples as in (A) were analyzed by western blotting using anti-Myc, anti-His, anti-Arm and anti-GST antibodies. Src64 and Src42, but not Mbt, were co-purified with GST-N-Cad(intra). (C) Western blot of GST-N-Cad(intra) pulldown samples was first probed with anti-phospho-tyrosine antibody (upper picture) and then re-probed with anti-GST. Shown is the overlay of both antibody signals verifying phosphorylation of GST-N-Cad(intra) in the presence of SRC proteins, but not of Mbt. (B,C) Shown are representative blots from three independent experiments. (D) Intensities of the upper and lower GST-N-Cad(intra) band for each lysate in (A) were quantified and the mean ratio was calculated from six to seven independent experiments. Co-expression of Mbt, but not of Src42 or Src64 resulted in a significant ratio shift.

The observed genetic interaction between *mbt* and *Src* on the one hand and the missing biochemical link between both proteins on the other hand, supported the idea that both kinases act in parallel pathways, which finally converge on N-Cad. To test for a potential effect of Mbt on N-Cad, stably transfected S2 cells expressing the GST-tagged intracellular domain of N-Cad [GST-N-Cad(intra)] alone or in combination with Myc-tagged Mbt were used. Western blot analysis of cell lysates detected two bands for GST-N-Cad(intra), which might be due to post-translational modifications or cleavage. Interestingly, in lysates from cells co-expressing GST-N-Cad(intra) and Mbt the upper band was consistently stronger in seven independent experiments (Fig. 7A, left and right lane). Quantification and calculation of the ratio between the upper and lower band verified a significant stabilizing effect of Mbt on the upper GST-N-Cad(intra) protein band (Fig. 7D). It remains an open question as to whether stabilization of the intracellular N-Cad domain by Mbt goes along with N-Cad/Arm complex stability. The stabilization or protection of GST-N-Cad(intra) by Mbt in cell culture could provide an explanation why Mbt promotes N-Cad AJ elongation during eye development. One possibility would be direct binding of Mbt to N-Cad. However, in the S2 cell culture system, Mbt was not co-purified with N-Cad(intra) in GST-pulldown experiments (Fig. 7B, right lane), arguing for additional, so far unknown molecules mediating the stabilizing effect of Mbt on N-Cad.

### N-Cad binds SRC and become tyrosine phosphorylated

Based on findings in other organisms, SRC phosphorylates N-Cad and influences N-Cad AJ stability (Leonard et al., 2013; Qi et al., 2006), *Drosophila* Src64 and Src42 might also directly affect N-Cad. In contrast to Mbt, co-expression of either Src64 or Src42 with GST-N-Cad(intra) in S2 cells had no effect on GST-N-Cad(intra) expression levels (Fig. 7A,D). Yet, GST-pulldown assays verified strong binding of N-Cad to Src64 whereas binding to Src42 was consistently weaker (Fig. 7B, middle lanes). For both SRC isoforms, this interaction resulted in tyrosine phosphorylation of GST-N-Cad(intra). The most prominent band detected with the pan-phospho-tyrosine antibody corresponds to the upper GST-N-Cad(intra) band, weaker signals match with the lower GST-N-Cad(intra) band as well as with Src64 and Src42 (Fig. 7C). The cell culture results match the finding *in vivo* that SRC kinase activity must be precisely regulated to allow elongation of N-Cad AJ (Fig. 5). Whether N-Cad phosphorylation in *Drosophila* affects N-Cad/Arm complex stability as observed in human cells (Qi et al., 2006) and which tyrosine sites are phosphorylated by Src64 and Src42 remain interesting questions for future studies. Nevertheless, *Drosophila* SRC proteins behave similarly to vertebrate SRC proteins with respect to N-Cad binding, phosphorylation and AJ destabilization.

## CONCLUSION

Mbt and SRC have opposite influences on N-Cad AJ during eye development. Mechanistically, one can envisage two different models. First, both kinases act independently on N-Cad AJ, Mbt as a positive and SRC as a negative regulator. In an alternative scenario, Mbt negatively regulates SRC to prevent its destabilizing effect on N-Cad AJ. The genetic interaction experiments under Mbt and SRC overexpression and loss of function conditions did not allow to favor one model. However, the lack of any direct biochemical link between Mbt and SRC rather indicated parallel signaling pathways which converge on N-Cad. Further support came from cell culture experiments, where Mbt enhances the stability of GST-N-Cad(intra) whereas SRC does not promote destabilization, although SRC binds and phosphorylates GST-N-Cad(intra). However, SRC's inability to destabilize N-Cad(intra) *in vitro* is in contrast to the observed disturbance of N-Cad AJ by overexpression of catalytically-active SRC. Therefore, it will be necessary to understand N-Cad dynamics in more detail in terms of synthesis, transport, membrane recruitment and degradation *in vivo*. To conclude, at least some aspects of Mbt signaling on N-Cad AJ regulation are probably independent from SRC activity.

## MATERIALS AND METHODS

### Cloning procedures and plasmids

To generate a genomic *mbt* rescue fly line, a genomic EcoRI fragment of the complete *mbt* sequence including 1410 bp upstream and 3526 bp downstream of the open reading frame was cloned into pW8 vector. The final vector was injected in *w^1118^* embryos. The insertion was mapped on the third chromosome and crossed into the *mbt^P1^* background (*mbt^P1^;P[gen-mbt]*, provided by Thomas Raabe, Würzburg, Germany).

For expression of Myc-tagged Mbt in S2 cells, the plasmid pMT-Myc-Mbt^wt^ was used (Menzel et al., 2008). To generate a His-tagged Src42 construct under the control of the metallothionein promoter (pMet-Src42-his), the pMet-PYO-Src42A-wt plasmid (Laberge et al., 2005) was used as a template to replace the C-terminal PYO-tag for a 6xHis-tag by the SLIM-PCR technique (Chiu et al., 2004). To create a His-tagged Src64 variant expressed under the control of the metallothionein promoter (pMet-Src64-his), the Src64 encoding sequence was amplified from pUChsneo-dSrc64B-wt (Kussick and Cooper, 1992; received from Mar Ruiz-Gomez, Madrid, Spain). Finally, Src42 was replaced by Src64 in the pMet-Src42-his plasmid-backbone.

### Fly stocks and genetics

Flies were kept at 25°C under a 12 h light, 12 h dark cycle unless otherwise noted. The following fly-lines were used: *mbt^P1^* (Melzig et al., 1998), *UAS-mbt^wt^*, *UAS-mbt^H19,22L^* and *UAS-mbt^K397M^* (encoding for wild-type, PBD defective and kinase dead Mbt; Schneeberger and Raabe, 2003). In all experiments *w^1118^* was used as wild type.

Other mutant alleles and transgenes used were: *Src42^26-1^* [also known as *Src42^−^*, protein-null and lethal mutant (Takahashi et al., 2005), received from Sol Sotillos Martín, Sevilla, Spain], *Src42^k10108^* [lethal P element insertion (Therrien et al., 1998), Bloomington Drosophila Stock Center (BL) #10969], *Src64^KO^* [protein-null (O'Reilly et al., 2006), received from Sol Sotillos Martín, Sevilla, Spain], *Src64^KG00213^* [weak, viable *Src64* allele (Djagaeva et al., 2005), BL#13646], *UAS-Src64* (also known as *UAS-Src64.C*, wild-type transgene, BL#8477), *UAS-Src42^16-2^* [wild-type transgene, (Pedraza et al., 2004), received from Stefan Luschnig, Münster, Germany], *UAS-Src42^CA^* [encoding for a constitutively active Src42 (Tateno et al., 2000), BL#6410], *ubi-ECad::GFP* [expresses GFP-tagged E-Cad under the ubiquitin-promotor, (Oda and Tsukita, 2001)].

The following Gal4-lines were used to express UAS-transgenes: *sev-Gal4* (*P[GAL4-Hsp70.sev]*, expression in the Sevenless pattern including photoreceptors R3 and R4 (Brand and Perrimon, 1993; Ray and Lakhotia, 2015), BL#2023), *gmr-Gal4* (expression in all cells behind the morphogenetic furrow, BL#1104).

For MARCM analysis (Lee and Luo, 1999), mitotic recombination between FRT sites was induced in female first- and second-instar larvae of the genotype *mbt^P1^,FRT19A/heat-shock-promotor-Flp,tubulin-promotor-GAL80,FRT19A;UAS-mCD8::GFP/+;ey^OK107^-Gal4/+* (Melzer et al., 2013) by two 1 h heat shocks (37°C). Homozygous *mbt^P1^* cells were detected by mCD8::GFP expression.

### Immunohistochemistry

Eye discs were dissected in PBS from wandering male third-instar larvae and directly fixed in PLP (75 mM lysin, 10 mM NaIO_4_, 2.8% paraformaldehyde in 30 mM sodium-phosphate buffer pH 6.8) for 20 min on ice unless otherwise noted. Eye discs were washed twice with PBT (0.3% TritonX100 in PBS) before blocking in 5% NGS [normal goat serum (NGS) in PBT] for 30 min at room temperature. Eye discs were incubated at 4°C overnight with the following primary antibodies: rat anti-N-Cad [1:25, Developmental Studies Hybridoma Bank (DSHB, DN-Ex#8)]; mouse anti-Armadillo (1:50, DSHB, N27A1), rat anti-E-Cad (1:100, DSHB, DCAD2), rabbit anti-Baz (1:500, a kind gift from Andreas Wodarz, Cologne, Germany), rabbit anti-GFP (1:1000, MoBiTec, A6455) or rabbit anti-Mbt (Schneeberger and Raabe, 2003). Following two washing steps in PBT, eye discs were incubated for 2 h at room temperature with secondary antibodies: donkey anti-mouse-Cy5 (Dianova); donkey anti-rat-Cy3 (Dianova); goat anti-rabbit-Alexa488 (MolecularProbes) all 1:100. After washing in PBT, eye discs were embedded in VectaShield (Vector Laboratories) and confocal images were recorded either with an Olympus Fluoview 1000 IX 81 or a Leica TCS SPE microscope.

### Image analysis and statistics

Confocal images were processed and analyzed using the ImageJ distribution FIJI (Schindelin et al., 2012). For the analysis of N-Cad AJ length between R3 and R4, ommatidia of the fifth row posterior to the morphogenic furrow were assessed according to the criteria in Fig. 2B. Data were plotted as percentage of measured ommatidia.

To obtain an independent value per eye disc, the median N-Cad AJ length was determined per eye disc. Several eye discs per genotype were analyzed. For statistical analysis the software R was used. Data were analyzed with the Wilcoxon-Mann-Whitney test from the coin-package followed by Bonferroni correction for multiple comparisons. Not significant results are marked by n.s. and statistical significance is stated by asterisks indicating the following *P*-values: *<0.05, **<0.01 and ***<0.001.

### Sequence comparison

Multiple protein sequences alignments were accomplished using Clustal Omega (Sievers et al., 2011).

### Cell culture, transfection and protein expression

S2 cells were maintained at 25°C in Schneider's *Drosophila* Medium (Biowest) supplemented with 10% fetal bovine serum (Biochrom AG), 2 mM L-glutamine (PAA) and 1% penicillin/streptomycin (PAA). Transfections of S2 cells were done with Cellfectin II Reagent (Invitrogen) according to the manufacturer's protocol. For stable transfection pCo-Hygro was added to the transfection mixture and afterwards cells were selected with 200 µg/ml Hygromycin (InvivoGen). Protein expression was induced by addition of 0.7 mM CuSO_4_ for about 20 h followed by cell lysis.

### Lysis and GST-pulldown assay

S2 cells were harvested by centrifugation at 1000× ***g***. Cell pellets were resuspended in lysis buffer (1% Nonidet-P40, 50 mM NaF, 150 mM NaCl, 2 mM EDTA, 50 mM Tris pH 7.8) supplemented with Complete (Roche) and PhosSTOP (Roche). The lysate was incubated at 4°C for 40 min with gentle shaking, centrifuged for 30 min at 16,000× ***g*** and 4°C, and the supernatant was used for further experiments.

To carry out GST-pulldown assay, lysates were incubated with Glutathione-Agarose (Machery-Nagel) for 4 h at 4°C with gentle shaking. Before elution, beads were washed three times with lysis buffer at 4°C. To elute proteins, beads were boiled in SDS-PAGE sample buffer.

### PAGE and western blot

Protein samples were separated using 10% SDS polyacrylamide gels and transferred to nitrocellulose, membranes were blocked with 5% dry milk in TBST (0.1% Tween20 in TBS). Primary antibodies were diluted in blocking solution: anti-Myc (1:2500, Santa Cruz Biotechnology, sc-40), anti-His (1:2500, Thermo Fisher Scientific, MA1-21315), anti-GST (1:20,000, Cell Signaling Technology, 26H1), anti-pTyr (1:1000, Santa Cruz Biotechnology, sc-508). Protein bands were detected with a ChemoCam (Intas) using HRP-coupled secondary antibodies (GE Healthcare) and ECL Prime Western Blotting Detection Reagent (GE Healthcare).

## Supplementary Material

Supplementary information
