# Supplementary material for: Mbt/PAK4 together with SRC modulates N-Cadherin adherens junctions in the developing Drosophila eye
Source: Biol Open. 2019 Mar 15;8(3):bio038406. doi: 10.1242/bio.038406 (PMC6451336; doi:10.1242/bio.038406)
Supplement: Supplementary information [file biolopen-8-038406-s1.pdf]

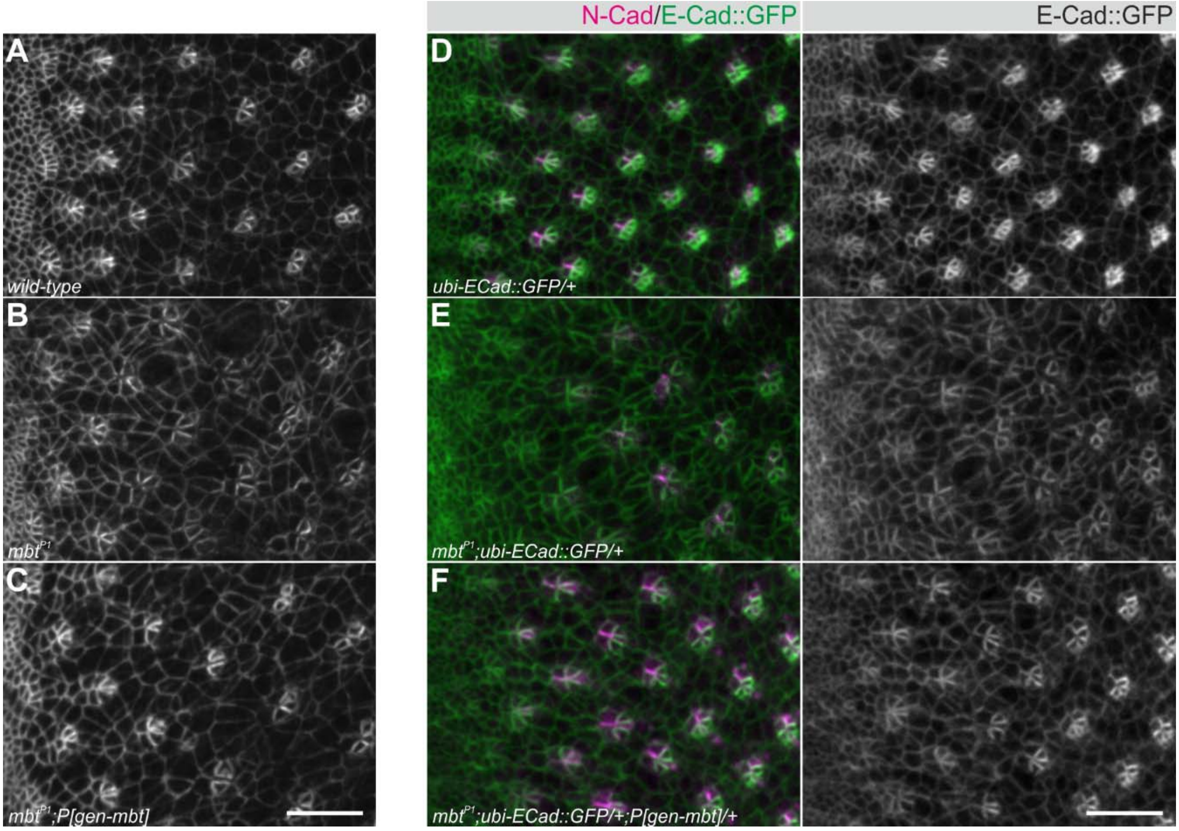

Figure S1 - **E-Cad accumulation at the R2 and R5 AJ is impaired in *mbt*^{P1} larval eye discs.** Image sections of eye discs posterior to MF of the genotypes wild-type (**A**), *mbt*^{P1} (**B**), *mbt*^{P1}*,P[gen-mbt]* (**C**), ubi-ECad::*GFP/+* (**D**), *mbt*^{P1},ubi-ECad::*GFP/+* (**E**) and *mbt*^{P1},ubi-ECad::*GFP/+,P[gen-mbt]/+* (**F**) stained for E-Cad (**A-C**) or GFP (green) and N-Cad (magenta) (**D-F**), respectively. Scale bars are 10µm and n>7 for each genotype.

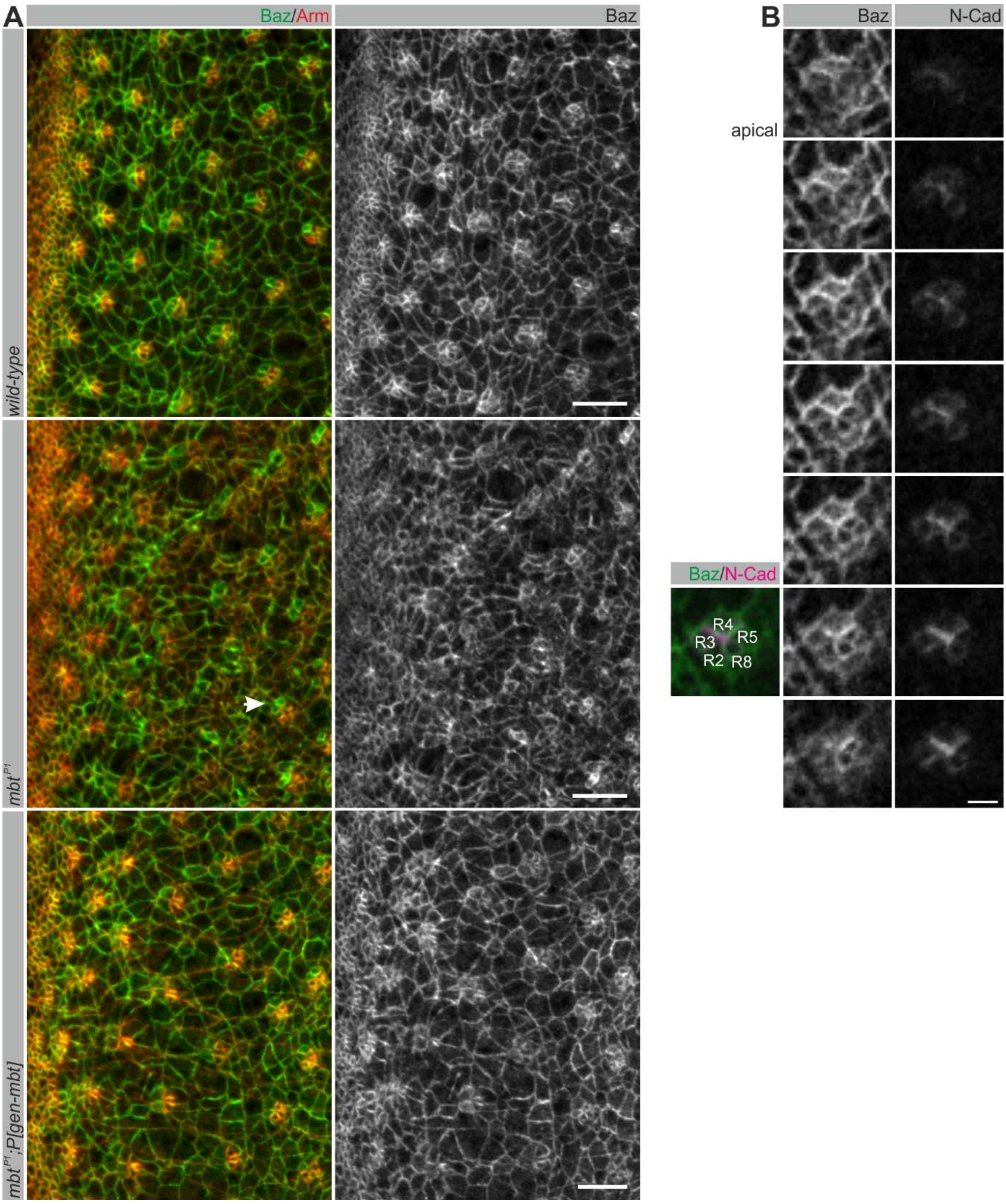

Figure S2 – Baz in *mbt^{P1}* larval ommatidia shows weak, more diffuse and uneven membrane localization. A) Image sections of eye discs posterior to MF of the genotypes wild-type (n=5), *mbt^{P1}* (n=10) and *mbt^{P1},P[gen-mbt]* (n=6) stained for Bazooka (green) and Arm (red). The arrowhead indicates elevated Baz in R4. Scale bars are 10µm. **B)** Image stack from apical to more basal sections of a wild-type row 5 ommatidium stained for Baz and N-Cad. Z-step size is 0.21 µm and scale bar is 2 µm.

Biology Open • Supplementary information

Biology Open (2019): doi:10.1242/bio.038406: Supplementary information

```
NCad_Dm       ---YNRRREAHIKYPGPDDDVRENIINYDDEGGGEDDMTAFDITPLQIPIG---------   2985
NCad_M        MKRRDKERQAKQLLIDPEDDVRDNILKYDEEGGGEEDQD-YDLSQLQQPDTVEPDAIKPV   804
NCad_H        MKRRDKERQAKQLLIDPEDDVRDNILKYDEEGGGEEDQD-YDLSQLQQPDTVEPDAIKPV   804
NCad_C        -KRRDKERQAKQLLIDPEDDVRDNILKYDEEGGGEEDQD-YDLSQLQQPDTVEPDAIKPV   810
                  ::.*:*:    .*:****:**::**:*****:*   :*:: ** *

NCad_Dm       GPMPPELAPMKMPIMYPVMTLMPGQEPNVGMFIEEHKKRADGDPNAPPFDDLRNYAYEGG   3045
NCad_M        GIRRLDERPIHAEPQYPVRSAAP-HPGDIGDFINEGLKAADNDPTAPPYDSLLVFDYEGS   863
NCad_H        GIRRMDERPIHAEPQYPVRSAAP-HPGDIGDFINEGLKAADNDPTAPPYDSLLVFDYEGS   863
NCad_C        GIRRLDERPIHAEPQYPVRSAAP-HPGDIGDFINEGLKAADNDPTAPPYDSLLVFDYEGS   869
              *    :  *::    *** :  * :  ::* **:*  * **.**.***:*.*  : ***.

NCad_Dm       GSTAGSLSSLASGTDDEQQEYDYLGAWGPRFDKLANMYGPEAPNPHNTELEL           3097
NCad_M        GSTAGSLSSLNSSSSGGDQDYDYLNDWGPRFKKLADMYGGGDD---------           906
NCad_H        GSTAGSLSSLNSSSSGGEQDYDYLNDWGPRFKKLADMYGGGDD---------           906
NCad_C        GSTAGSLSSLNSSSSGGEQDYDYLNDWGPRFKKLADMYGGGDD---------           912
              ********** *.:.. :*:****. *****.***:***
```

Figure S3 - **Conserved SRC phosphorylation sites within the N-Cad cytoplasmic domain from different organisms.** Sequence alignment from human (H), mouse (M), chicken (C) and *Drosophila* (Dm) N-Cad cytoplasmic domains. The tyrosine phosphorylation site relevant for β-Catenin dissociation (Y860 in human, Y3042 in *Drosophila*, marked in green) is conserved across phyla. Besides, human SRC is able to phosphorylate further tyrosine residues (marked in blue), all but one are conserved in mouse, chicken and *Drosophila* N-Cad.

Biology Open • Supplementary information

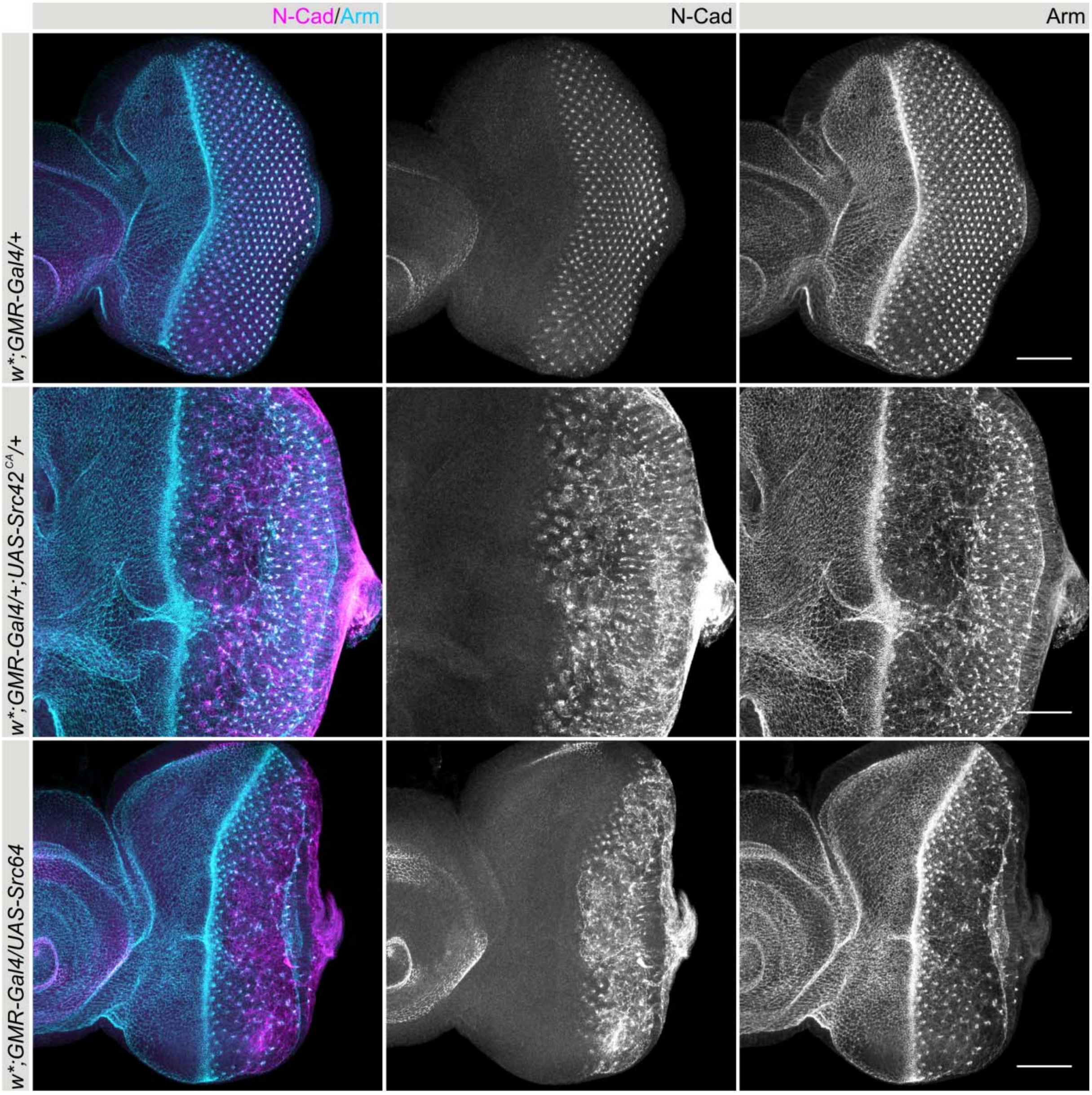

Figure S4 - Elevated expression of Src64 or constitutively active Src42^{CA} at 25°C disrupts eye development. Eye discs from animals expressing either *UAS-Src64* or *UAS-Src42^{CA}* under the control of *gmr-Gal4* stained for N-Cad (magenta) and Arm (cyan). Eye discs from *gmr-Gal4/+* animals were used as control. Scale bar is 50µm.

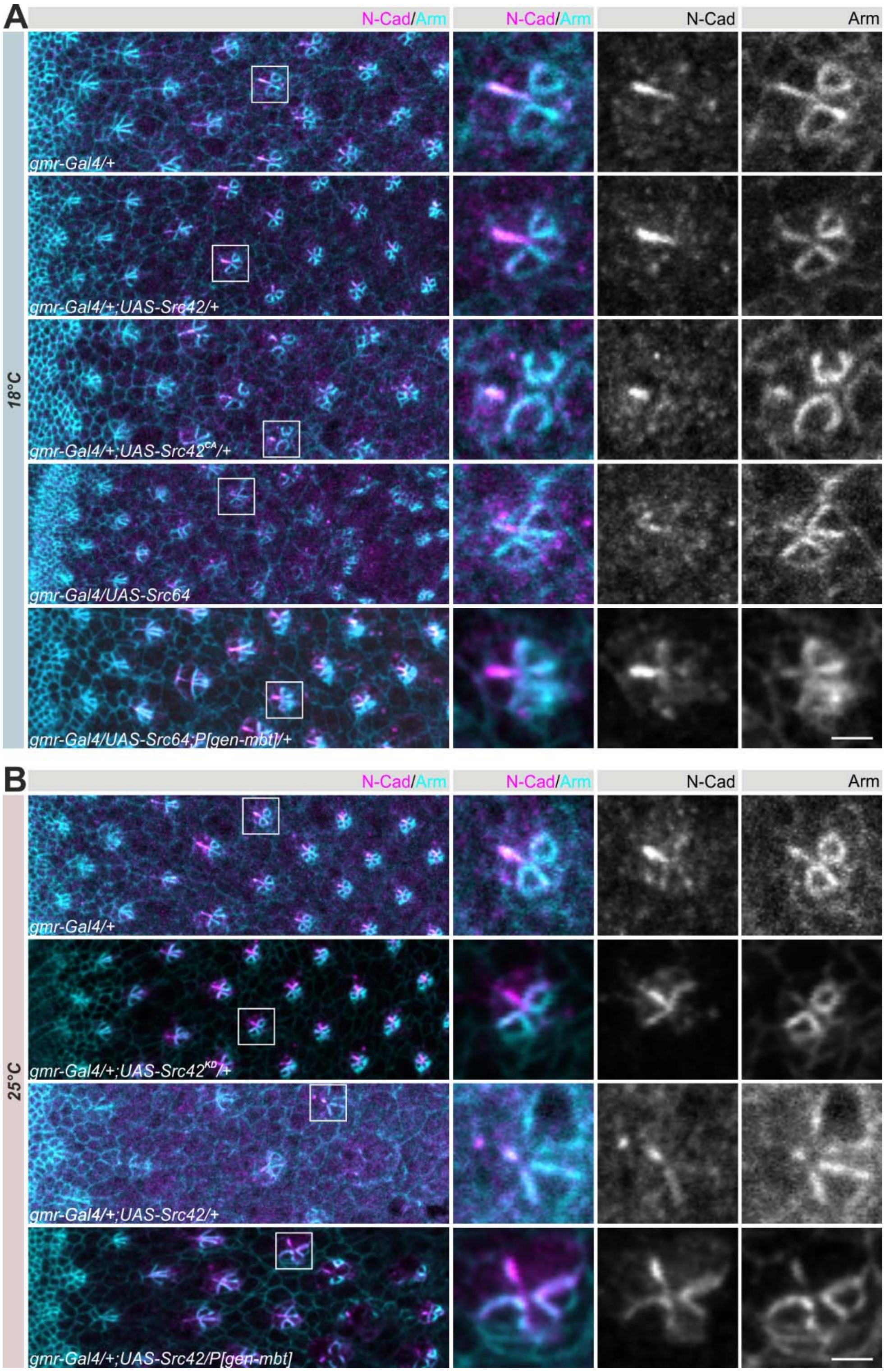

Figure S5 - Elevated expression of SRC isoforms shorten the N-Cad AJ between photoreceptors R3 and R4. Larval eye discs and representative row 5 ommatidia from animals expressing *UAS-Src64*, *UAS-Src42*, *UAS-Src42*^{KD} or *UAS-Src42*^{CA} under *gmr-Gal4* control were stained for N-Cad (magenta) and Arm (cyan). To test for genetic interaction, *UAS-Src64* and *UAS-Src42*^{CA} were also analyzed in combination with *P[gen-mbt]*. *gmr-Gal4/+* are used as control. Animals grew up at 18°C **(A)** or 25°C **(B),** respectively. Scale bars are 2µm.

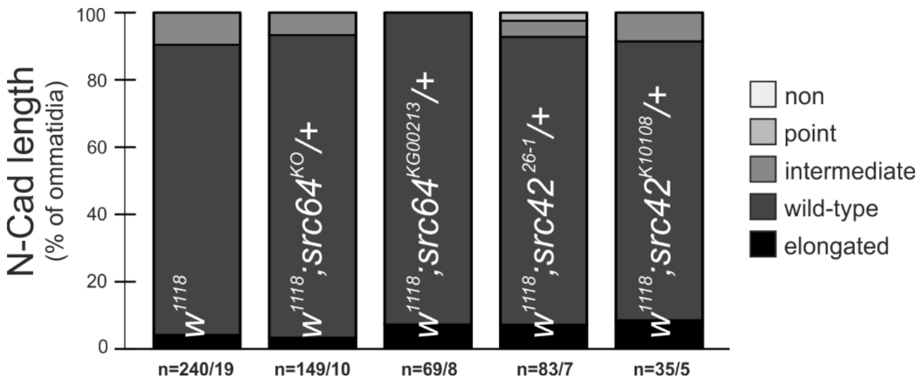

Figure S6 – **Heterozygous *Src* mutations do not change N-Cad AJ length.** Quantitative analysis of the N-Cad AJ length from ommatidia heterozygous for the indicated *Src42* or *Src64* alleles. N-Cad AJ were classified according to **Fig. 2B**.

Biology Open • Supplementary information

```
                                  PBD
A  Mbt_Dm     MFSKKKKKPLISMPSNFEHRVHTGFDKRENKYVGLPLQWASIVGNNQILKSSNRPLPLVD  60
   PAK4_M     MFGKKKKRVEISAPSNFEHRVHTGFDQHEQKFTGLPRQWQSLIE-----ESARRPKPLID  55
   PAK4_H     MFGKRKKRVEISAPSNFEHRVHTGFDQHEQKFTGLPRQWQSLIE-----ESARRPKPLVD  55
              **.*:**:   ** *************::*:*:.*** ** *::      :*:.** **:*

B  SRC64_Dm   MGNKCCSKRQD-----QELALAYPT  20
   SRC42_Dm   MGNCLTTQKGEPDKPADRIKLDDPP  25
   SRC_H      ----MGSNKSKPKDASQRRRSLEPA  21
   SRC_M      ----MGSNKSKPKDASQRRRSLEPS  21
                  :.: .     :.     *
```

Figure S7 – The PAK4 RPKP motive is changed to RPLP in Mbt and SRC S17 is absent in *Drosophila*. A) Sequence alignment of N-terminal PAK4/Mbt sequences from human (H), mouse (M) and *Drosophila* (Dm). High similarity is observed in the PBD, but the RPKP peptide (blue) is different in Mbt. **B)** Alignment of N-terminal SRC sequences from human (H), mouse (M) and *Drosophila* (Dm). The predicted S17 phosphorylation site (green) in human and mouse SRC is absent in *Drosophila* Src64 and Src42.

Biology Open • Supplementary information
